# School Children’s Sorrows

**Published:** 1861-04

**Authors:** 


### SCHOOL CHILDREN'S SORROWS,

PARTICULARLY those who go to public school. Of all the stupid creatures who walk this earth, they excel, who having the means to send their children to private school, and to control their times of study, do yet allow them in these debilitating spring months, to spend from six to eight hours in the putrefying air of an apartment, where a dozen and upwards are breathing each other's breaths over and over again, and then allow them to come home with a load of books, a foot or two high, to be pored over for a greater part of the time until next morning. But there is one good thing connected with such a management, three out of four of such children will die before they will have a chance of perpetuating their enfeebled brains and bodies, to be a curse to themselves, or a charge upon the charities of society.

We plead here for the little ones of that large class of more useful citizens who, working for a living, can not afford to pay two hundred dollars a year for each child sent to the private schools of this city, and who feel glad of the privilege of being able to send them, without cost for books or tuition, to our public schools, and which are as far superior, in New-York City, to the private schools, for all that is systematic, prompt, accurate, and thorough, as one can well imagine. We have had personal means of observation as to the patience, the fidelity, and conscientiousness of the teachers of New-York, especially of those connected with the Twelfth and Twentieth street schools for girls. At the same time, we do occasionally feel, perhaps when in an indigo condition, or when some east wind is blowing, or the upper house is in a state of insubordination, from sewing and patching and darning until past eleven the night before, that it admits of debate whether or not it would not be better to razee every public-school building to its foundation, provide all the young ladies who are teachers with good, thrifty, handsome husbands, and turn the children out to grass, literally, because an ignorant healthy young woman is worth more to the government and worth more to her husband when she gets one, than a cart-load of educated skeletons, or bags of bones, so thin and skinny, and tottering and frail, that literally a breath of air puts them off their feet, and sends them to bed. Young man, be advised while we think of it, if you want to be happy in married life; if you want to experience to its full, the lusciousness of the wedded state, the snow-white table-spread, the cheerful hearth, the cozy fireside and the blest reünion of wife, children, and friends, three times a day around a well-spread board, take for a wife a healthy girl, who had a good mother, but could not write her name or read a line, nor had a dime for dower, in preference to a young woman who has graduated in music, in dancing, in grammar, philosophy, and French, an heiress though she be, and of a position enviable enough, but after all has no physical health; come to think of it, we will just mention parenthetically, even if she have as good health as the other, choose the poor girl, for the simple financial reason, that by the time you have raised a family of children, the rich girl will have spent upon herself and on her children, and on her style of living, and have allowed to go to waste, by not stooping to attend to the vulgar employment of seeing to her domestic affairs, her pantries, her bins, and her tea-chest, more than she ever brought from her father; while the poor girl, of a good mother, will have saved as much, with a wealth of domestic enjoyment thrown in. These are beyond question, general truths; but it is not likely that one young man in a hundred will properly appreciate them. It is our private opinion, that an "educated" wife, like an educated negro, is in proportion unfitted for being happy in her place, unless the grace of God is predominant. This is a sentiment of infinite practical moment. The more educated a woman is, the more unlikely is she to make a good wife, unless true religion pervades her whole character. There are exceptions perhaps. This is not so far off the subject of public schools, as the reader may at first imagine; for it corroborates the general idea, that it is better to forego education than to sacrifice the bodily health.

The Hub of the Universe ordered some months ago, that no scholar in the public schools should have any lessons to learn out of school-hours. The New-York School Commissioners, made up in part of grocery-keepers, liquor-dealers, and ignoramuses, thought to improve on this idea, and ordered that there should be no lessons out of school-hours beyond what could be learned in an hour. The moment we laid our eyes on the ordinance, we saw it was an abortion; and yet the editorial fraternity bounded off in a universal jubilation. Who was to be the judge of what a child can learn in an hour? The capacities of children vary in a most extraordinary manner. The teacher is apt to estimate from what the best scholars can do, and inclines to the idea, that all could do as well as those at the head of the class, if they were not idle. But to a fact: we know a family which sends two daughters to a public school, within half a mile of Union Square; they leave home at half-past eight, and return at half-past three, a duration of seven hours. By four o'clock, they have eaten their dinners, and instead of going out to play, they have lessons which can not be mastered thoroughly by sitting up until nine o'clock, and waking by six next morning. The lessons given on Friday afternoon are such, "because you have a good long holiday," that they have had to give up the Sunday-school, and study a large portion of Sunday, in order to make a proper preparation for Monday morning. Of course there is no time for music-lessons, and, what some parents consider a great privation, for dancing-school. It is certainly known that no child can become moderately well versed in piano music, unless put to it before ten years old. The consequence is, that the public-school children must either neglect their school-lessons or grow up ignorant of music, deprived of all the advantages of a well-conducted Sunday-school, and are taught practically the constant perversion of the proper uses of the Sabbath-day. This view of the case is not a whit short of horrible, and we vouch for the literal truth of every syllable here penned. But who cares for the children of poor people? what business have they to be thumping on the piano, or jumping Jim Crow and "polking" about to the sound of the fiddle? But why are children thus pushed from seven years of age to seventeen? It is a good berth for a young lady to have a salary of from three to eight hundred dollars a year. She retains it by the showing she is able to make in bringing the children forward. The more a child can learn in the shortest time, the more capable is the teacher considered to be; this, that teacher knows, and without intending to murder the children, but to bring them on rapidly, she overlooks the actual facts of the case, the brightness of the pile of five hundred dollars for ten months' work, throwing every thing else in the shade, reason, humanity, and life itself. From three to four different lessons are given out to the public-school children every afternoon, and if a single question is missed, it is considered a "failure," and a failure is a disgrace to be punished by being kept in a half or a whole hour after a murderous endurance of the preceding six. But nothing better could be expected from a company of grog-sellers, shoulder-hitters, and bar-room loafers and politicians, such being the character of too many of the men who have a vote in the management of the public schools of this city. In one of the wards at the last November election, a gentleman of high social position, of travel, and superior culture, a patron of education, did not receive three hundred votes out of fifteen hundred for a school office, an honorable "furriner" winning the race by all odds. While this is going on, the editors of this city, "civil and religious," have not a word for the abatement of nuisances so fearful, but are head and ears in political or sectarian controversies, stirring up the people to greater political differences and to greater sectarian animosities. This is "the progress" of the nineteenth century. On the other hand, parents are so immersed in money-getting, that they give no heed to these things. Death alone, brings them to their consciousness. "I see it now," said a retired merchant to us, not long ago as he gazed on the emaciated form of his dead daughter, just graduated at eighteen, his only child! alluding to her acknowledged over-application, in her ambition to maintain herself at the head of the class.

"Father," said a bright-eyed little girl of twelve in our hearing to-day, "the girls in our class who miss most, study their lessons all the time from recess until next morning." There is fearful meaning in this, that in their efforts to master their lessons in the exhausted condition of the brain in the afternoon, after a six hours' application, the mind fails to work, and an inextricable confusion of intellect prevents any clear conception of one thing from another. Can any heart fail of sympathy for the willing but vain efforts to comprehend their studies? No child should be allowed to take a book home from public school; the teacher ought to be summarily dismissed who failed to make the child master the lesson to-day, in school-hours, which is to be recited to-morrow, and by half-past four, each child should be in the Central Park, breathing its pure air, drinking in its beauteous greens, loitering through its Ramble, rollicking along its Mall, or promenading its tortuous paths in search of some tiny flower of spring, or moss, or leaf, or budding twig.

Will the editors of this city take up the subject, or shall it still be that over half of all who die in New-York City, shall not reach their seventeenth year ! ! !

